# Supplementary material for: Heating-mediated purification of active FGF21 and structure-based design of its variant with enhanced potency
Source: Sci Rep. 2023 Jan 18;13:1005. doi: 10.1038/s41598-023-27717-x (PMC9849446; doi:10.1038/s41598-023-27717-x)
Supplement: Supplementary file 1 — Supplementary Figures. [file 41598_2023_27717_MOESM1_ESM.docx]

**Supplementary Information**

**Heating-mediated purification of active FGF21 and structure-based design of its variant with enhanced potency**

Ye-Eun Jung^1,7^, Kyeong Won Lee^2,7^, Jae Hyun Cho^3,7^, Da-Woon Bae^1^, Bo-Gyeong Jeong^1^, Yeon-Ju Jung^1^, Soo-Bong Park^1^, Young Jun An^2^, Kyungchan Kim^3^, Ga Seul Lee^4^, Lin-Woo Kang^5^, Jeong Hee Moon^4^, Jung-Hyun Lee^2^, Eun-Kyoung Kim^3,6, *^, Hyung-Soon Yim^2, *^, and Sun-Shin Cha^1, *^

^1^ Department of Chemistry & Nanoscience, Ewha Womans University, Seoul 03760, Republic of Korea

^2^ Marine Biotechnology Research Center, Korea Institute of Ocean Science and Technology, 385 Haeyang-ro, Busan 49111, Republic of Korea

^3^ Department of Brain Sciences, Daegu Gyeongbuk Institute of Science and Technology, Daegu 42988, Republic of Korea

^4^ Disease Target Structure Research Center, Korea Research Institute Bioscience and Biotechnology (KRIBB), Daejeon, 34141, Republic of Korea

^5^ Department of Biological Sciences, Konkuk University, Seoul, 05029, Republic of Korea

^6^ Neurometabolomics Research Center, Daegu Gyeongbuk Institute of Science and Technology, Daegu 42988, Daegu Republic of Korea

^7^ These authors contributed equally to this work.

^*^ Corresponding authors: [chajung@ewha.ac.kr](mailto:chajung@ewha.ac.kr) (S.-S. Cha); [yimh@kiost.ac.kr](mailto:yimh@kiost.ac.kr) (H.-S. Yim); [ekkim@dgist.ac.kr](mailto:ekkim@dgist.ac.kr) (E.-K. Kim)

Table of contents

**Supplementary Information**

Fig. S1. Observation of turbidity 3

Fig. S2. Structure-based sequence alignment among human FGFs 4

Fig. S3. Time-dependent plotting of OD_600_ at 75 ºC 5

Fig. S4. SDS-PAGE analysis of the Trx-FGF21 before (-) and after (+) heating 6

Fig. S5. DLS analysis of nFGF21 and htFGF21 7

Fig. S6. SDS-PAGE analysis of FGF21 purified with and without heating. 8

Fig. S7. The structure of the C-terminal loop of human FGF21 in complex with β-klotho 9

Fig. S8. Sequence alignment of the C-terminal loops (residues 193-209 in FGF21) of mammalian FGF21s 10

Fig. S9. Sequence alignment of mammalian β-Klothos 11

Fig. S10. SPR sensorgrams of FGF21s 12

Fig. S11. Heat map plot of predicted binding affinity for MHC class I alleles 13

Fig. S12. Heat map plot of predicted binding affinity for MHC class II alleles 14

Fig. S13. Prediction for B cell epitope 15

Fig. S14. Uncropped images of SDS-PAGE showed in Fig. 1A 16

Fig. S15. Uncropped images of Western-blot analyses showed in Fig. 4B and 6B 17

**Supplementary Information**

**
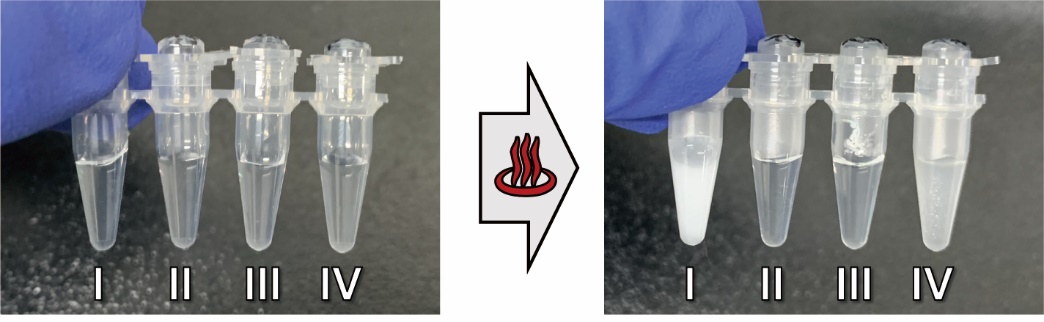
**

**Fig. S1. Observation of turbidity.** The photos show the turbidity of protein solutions in 0.2 ml tubes before (left) and after (right) heating at 100℃. I, II, III, and IV represent FGF2, the wild type FGF21, core-FGF21, and core-FGF21-LW, respectively.


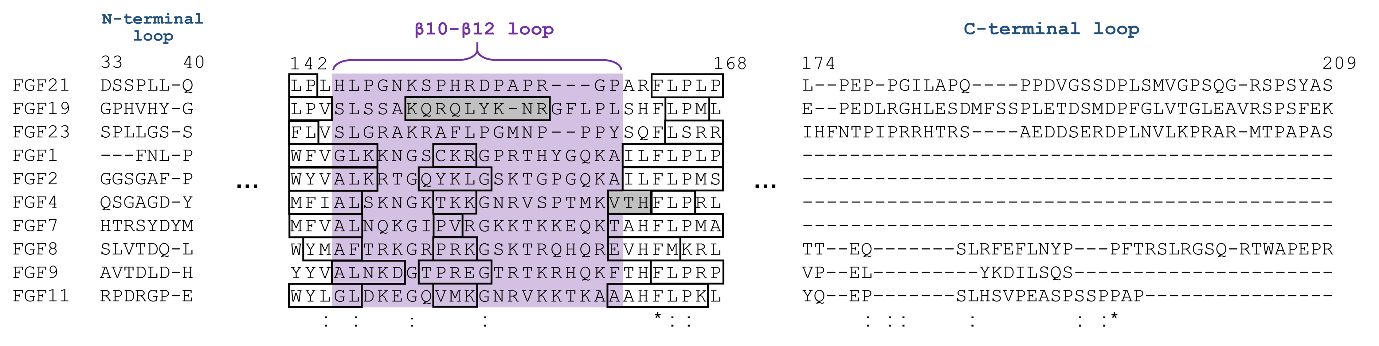


**Fig. S2. Structure-based sequence alignment among human FGFs.** Gray boxes indicate α-helices and empty boxes do β-strands. The region corresponding to the β10-β12 loop of FGF21 is shaded in violet. Dashes represent gaps introduced to optimize the alignment. The asterisks and colons indicate identical amino acid residues and conserved substitution, respectively.


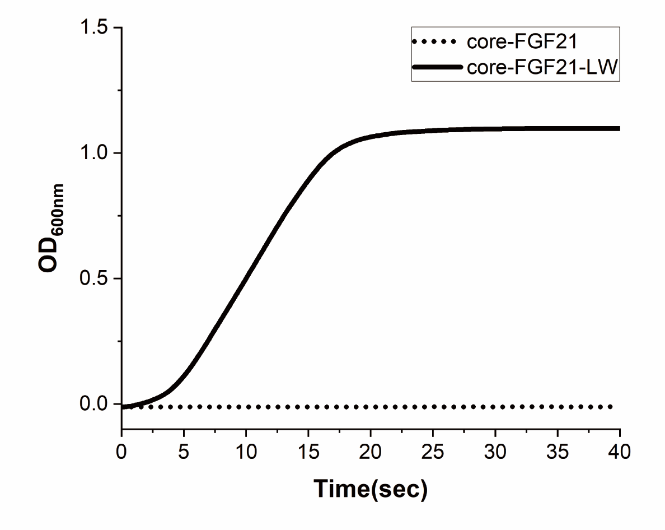


**Fig. S3.** **Time-dependent plotting of OD_600_ at 75 ºC.**


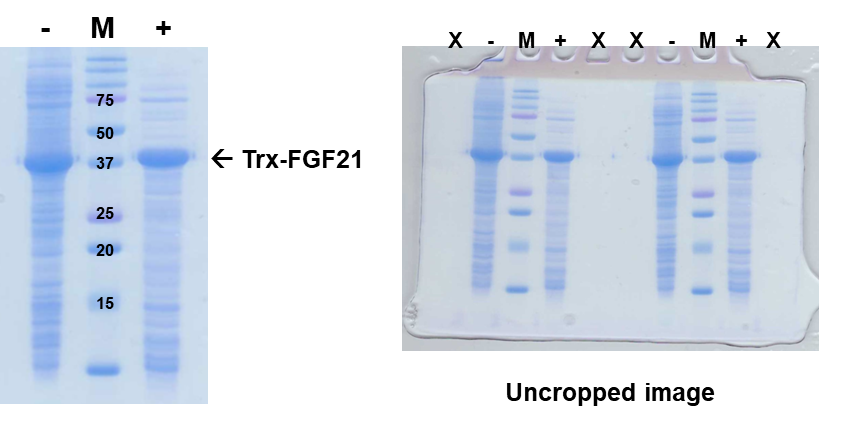


**Fig. S4. SDS-PAGE analysis of the Trx-FGF21 before (-) and after (+) heating.** M represents protein markers whose sizes are given in kDa on the band. Arrow points out the position of target protein.


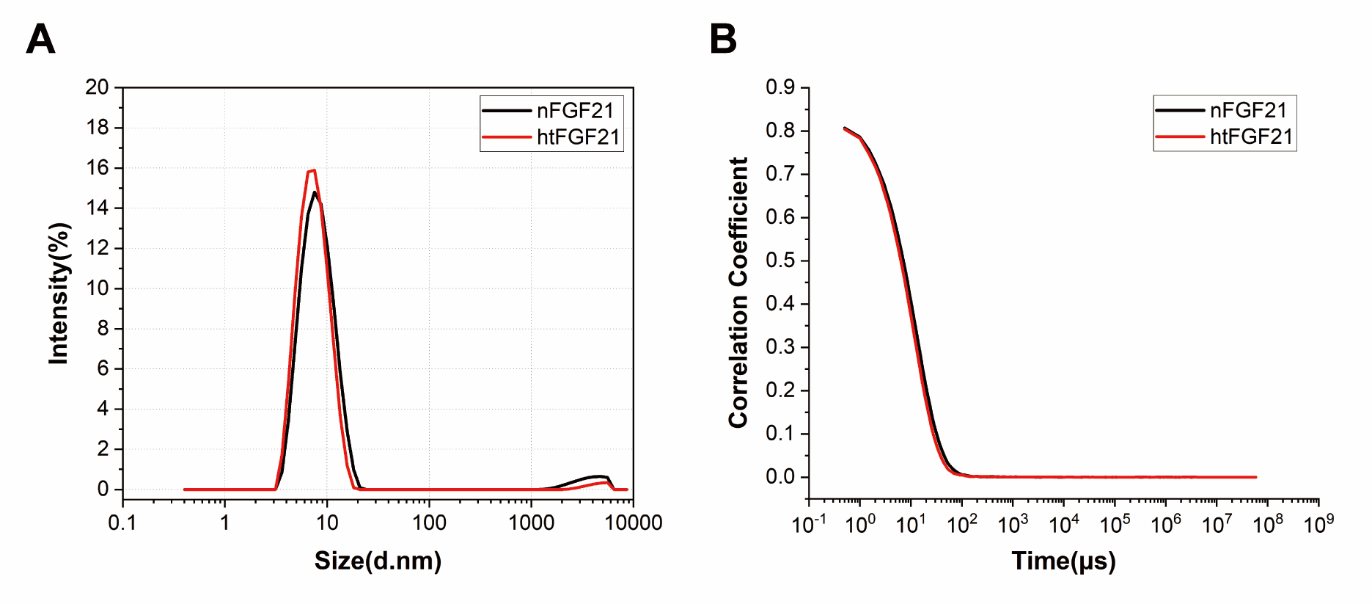


**Fig. S5. DLS analysis of nFGF21 and htFGF21.** (A) Comparison of the size distribution histograms between nFGF21 and htFGF21. (B) Raw data of autocorrelation functions detected by DLS. FGF21 purified through heat treatment is represented by htFGF21 while nFGF21 refers to FGF21 purified without heating.

**
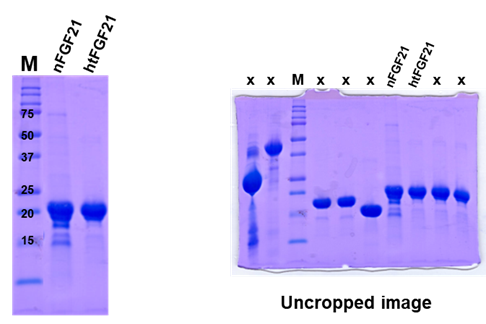
**

**Fig. S6 SDS-PAGE analysis of FGF21 purified with and without heating.** M represents protein markers whose sizes are given in kDa on the band. FGF21 purified through heat-treatment is represented by htFGF21 while nFGF21 refers to the protein purified without heating.


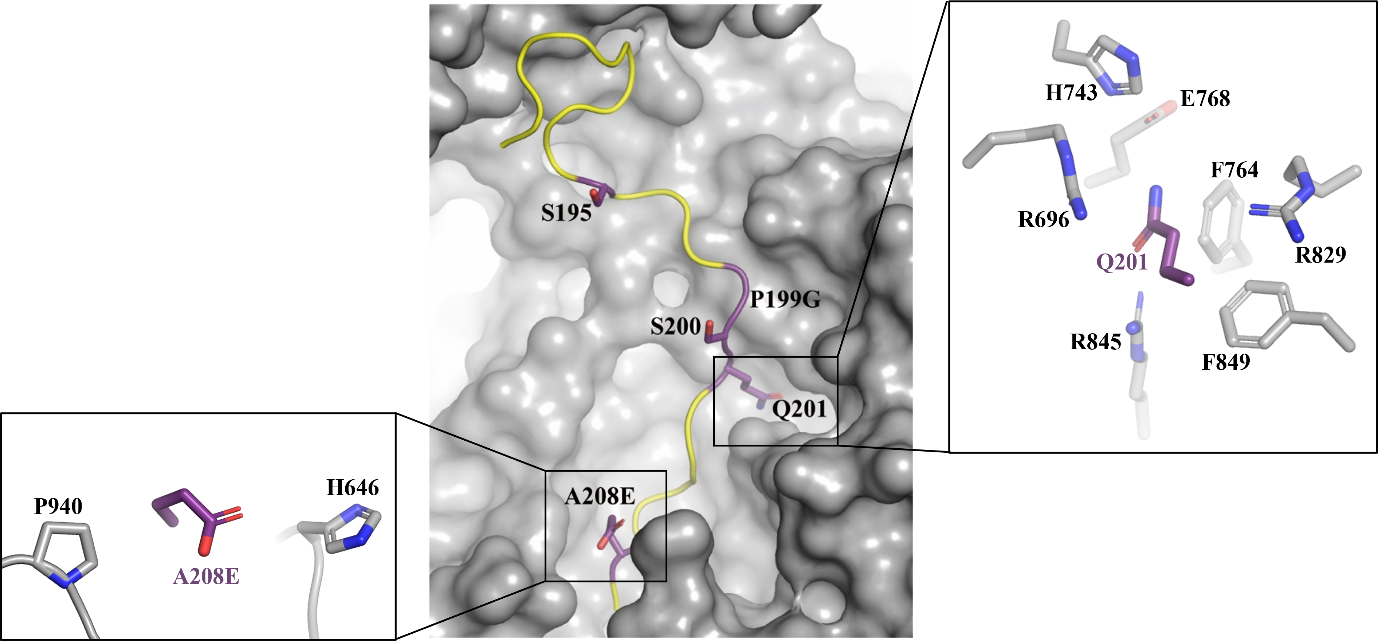


**Fig. S7. The structure of the C-terminal loop of human FGF21 in complex with β-Klotho (PDB code: 5VAQ) in which the C-terminal loop contains P199G and A208E mutations.** The C-terminal loop of FGF21 is shown in yellow cartoon with five mutation sites in purple sticks. β-Klotho is shown as gray sticks and surface representation. Nitrogen and oxygen atoms in sticks are colored in blue and red, respectively.


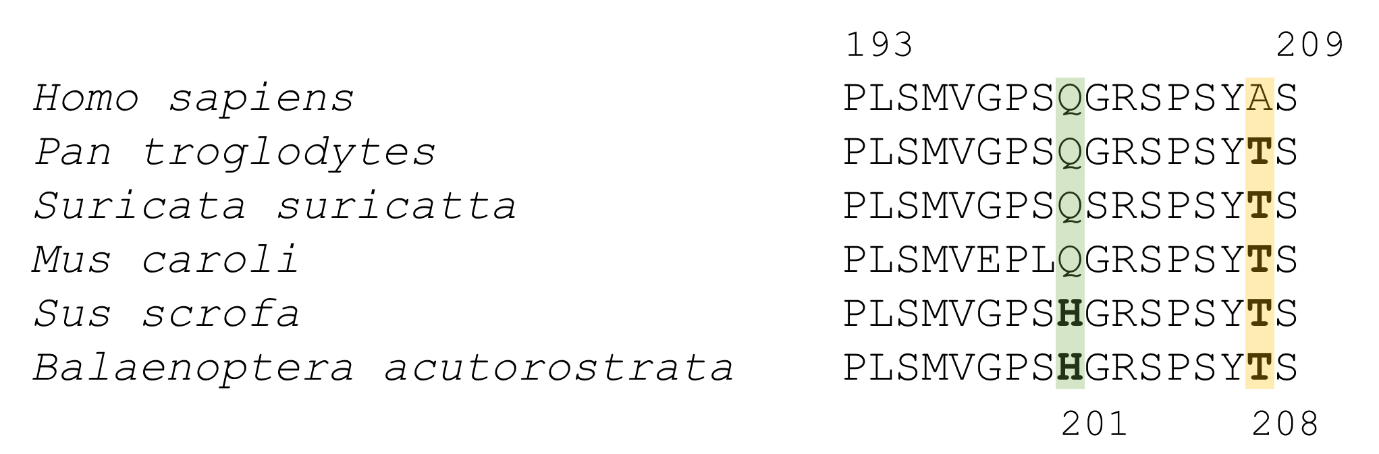


**Fig. S8. Sequence alignment of the C-terminal loops (residues 193-209 in FGF21) of mammalian FGF21s.** Amino acids at positions 201 and 208 are colored in green and yellow, respectively. Histidine and threonine at positions 201 and 208 are shown in bold. The GenBank accession numbers are as follows: NP_061986.1 (*Homo sapiens*), XP_016791946.1 (*Pan troglodytes*), XP_029780315.1 (*Suricata suricatta*), XP_021024694.1 (*Mus caroli*), NP_001156882.1 (*Sus scrofa*), XP_007168312.1 (*Balaenoptera acutorostrata scammoni*).


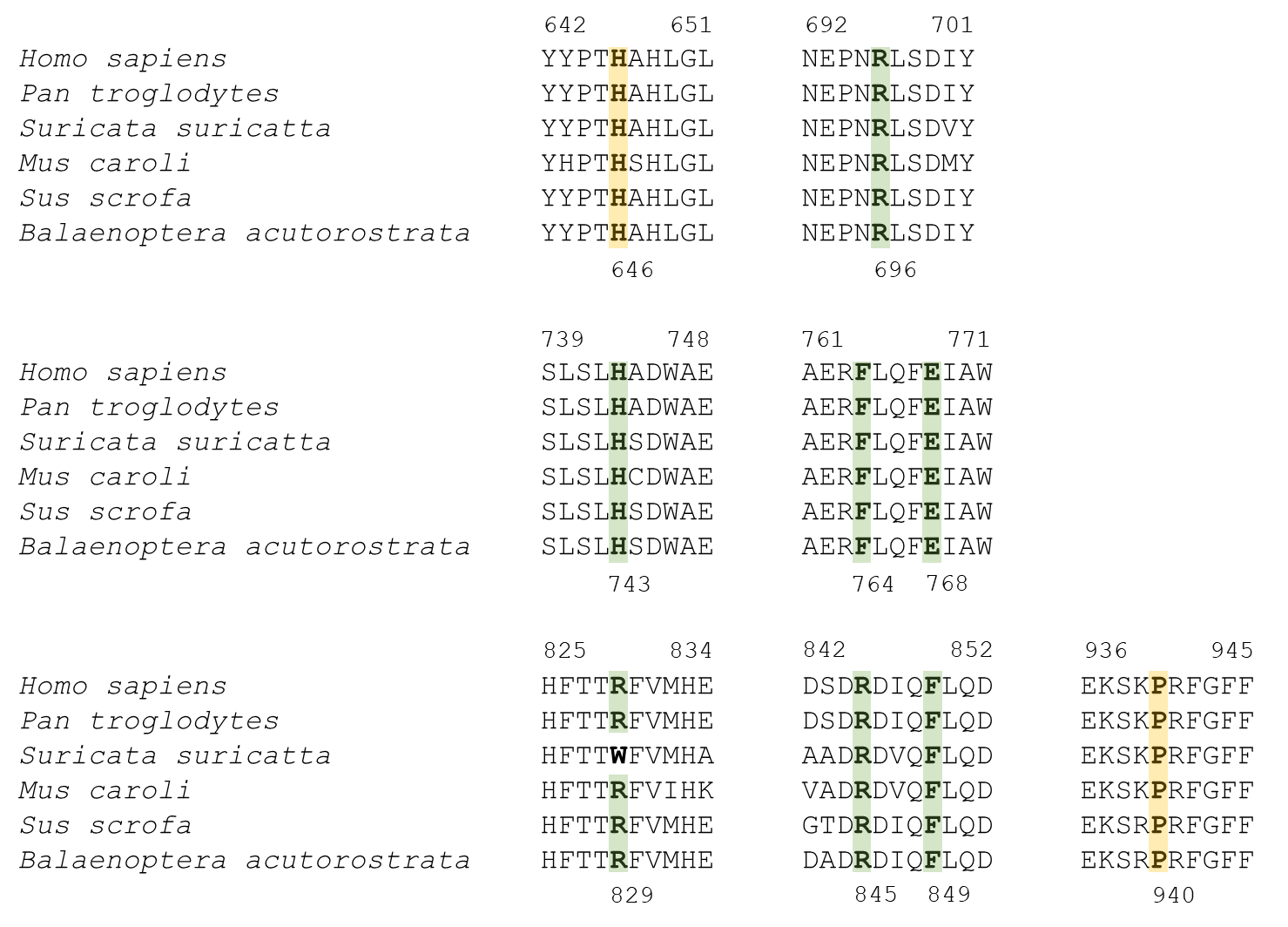


**Fig. S9. Sequence alignment of mammalian β-Klothos.** Amino acids interacting with residues at positions 201 and 208 of FGF21 are colored in green and yellow, respectively. They are shown in bold. The GenBank accession numbers are as follows: NP_783864.1 (*Homo sapiens*), XP_526550.1 (*Pan troglodytes*), XP_029803081.1 (*Suricata suricatta*), XP_021018417.1 (*Mus caroli*), XP_003482415.3 (*Sus scrofa*), XP_007178874.1 (*Balaenoptera acutorostrata scammoni*).


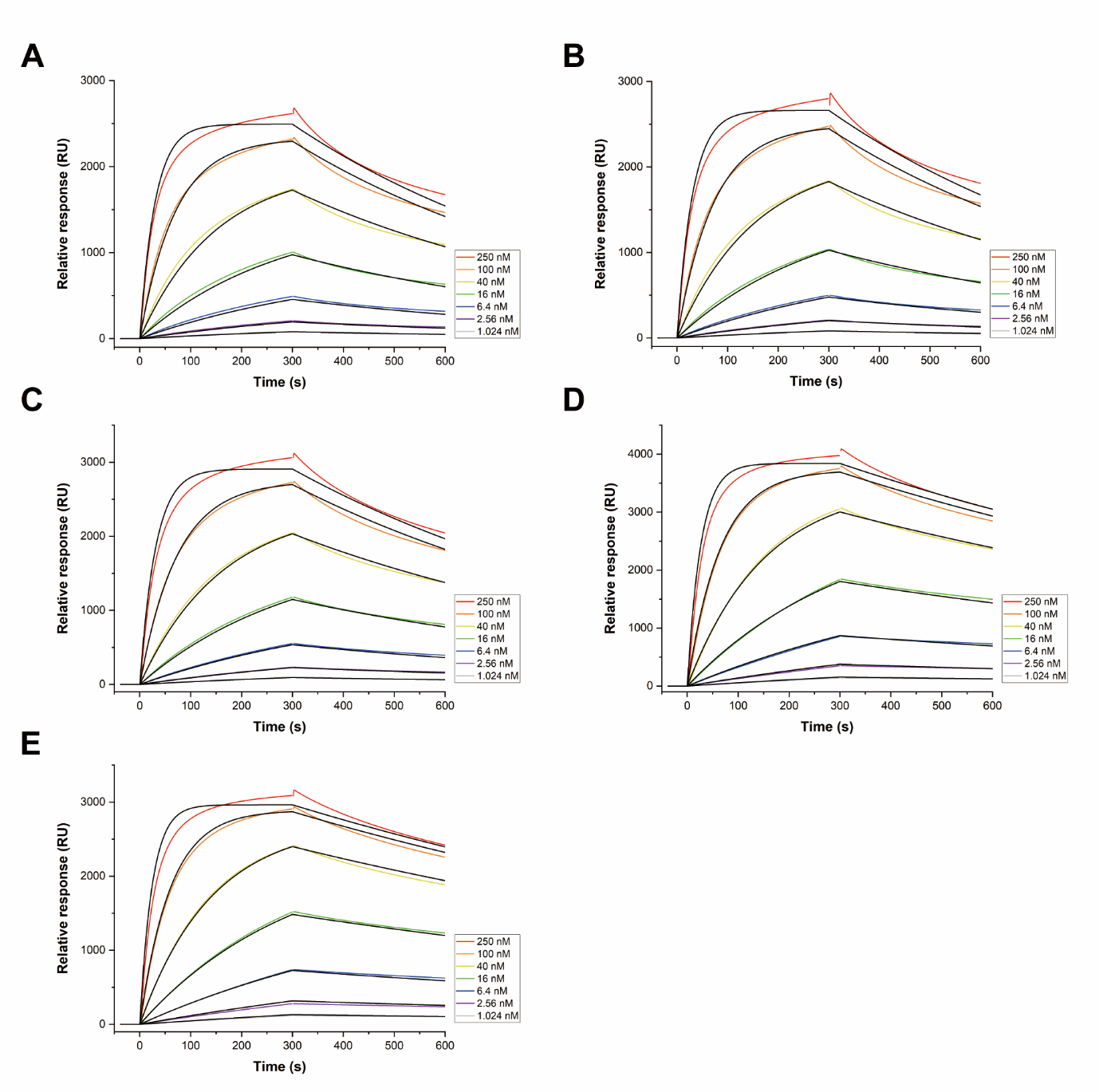


**Fig. S10. SPR sensorgrams of FGF21s.** The sensorgrams indicate that β-Klotho bound to nFGF21 (A), htFGF21 (B), htM2 (C), htM3 (D), and htM5 (E). One of three independent experiments is shown. The different concentrations of β-Klotho are represented using different colored lines as shown in the right box. Black lines are fitted curves.


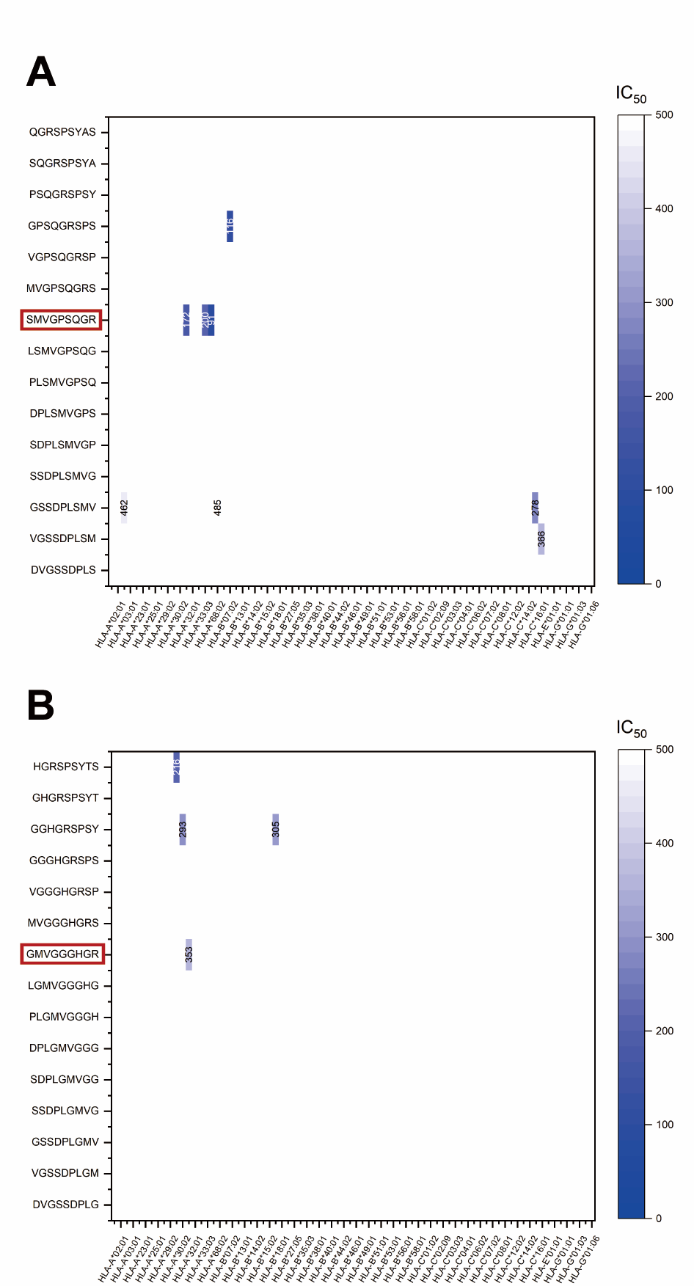


**Fig. S11. Heat map plot of predicted binding affinity for MHC class I alleles.** Each row indicates 9-mer linear peptides from wild type FGF21 (A) and M5 (B). The column represents different HLA variants. The IC_50_ values below 500 nM are shown by color gradient (blue to white) with labels. The “SMVGPSQGR” and “GMVGGGHGR” peptides of the wild type and M5 are marked with red squares.


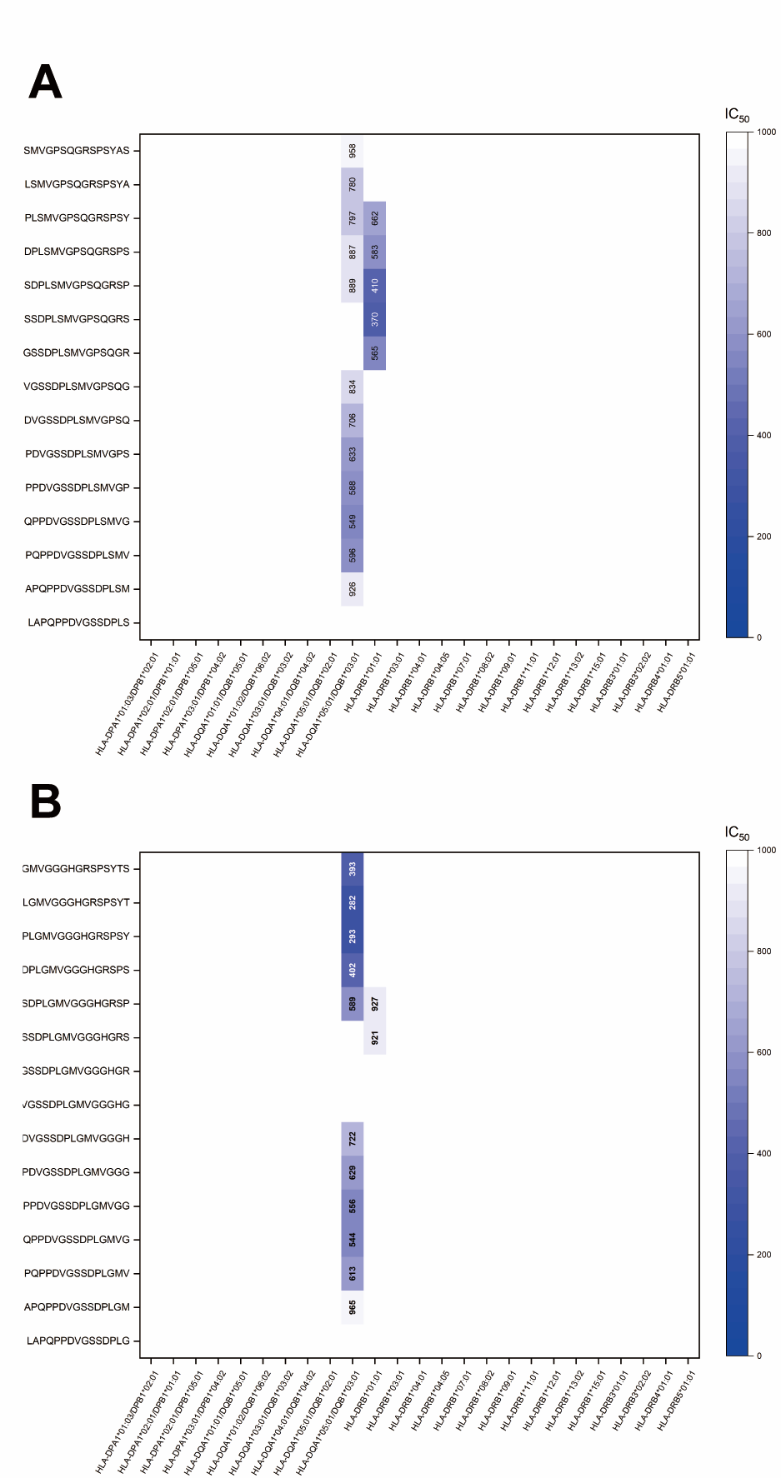


**Fig. S12. Heat map plot of predicted binding affinity for MHC class II alleles.** Each row indicates 15-mer linear peptides from wild type FGF21 (A) and M5 (B). The column represents different HLA-DP, -DQ, and -DR variants. The IC_50_ values below 1000 nM are shown by color gradient with labels.


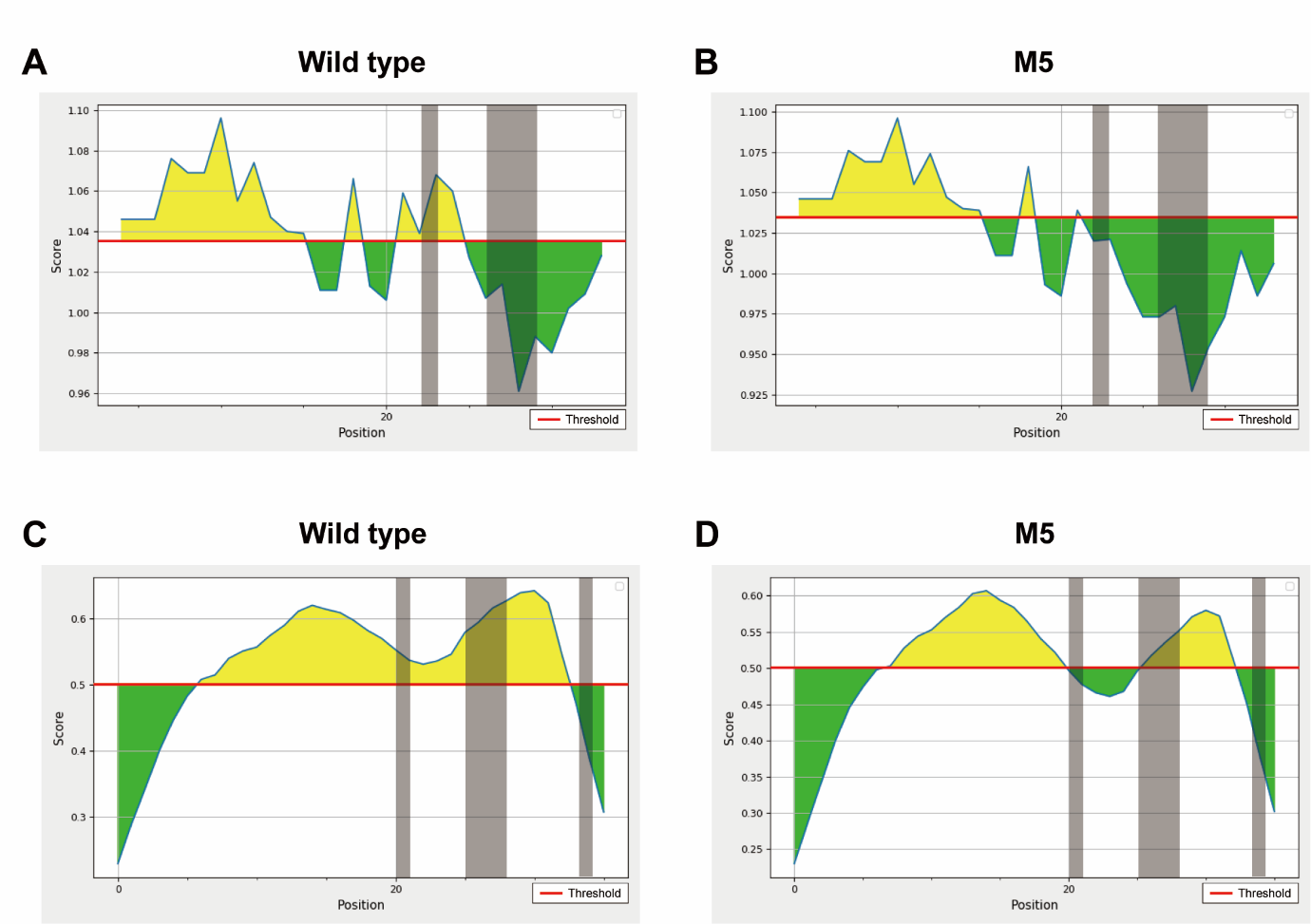


**Fig. S13. Prediction for B cell epitope.** Kolaskar and Tongaonkar antigenicity prediction for the wild type FGF21 (A) and M5 (B) (Threshold = 1.035; red line), Bepipred linear epitope prediction for the wild type FGF21 (C) and M5 (D) (Threshold = 0.5; red line). An x-axis indicates a sequence of the C-terminal loop (residues 174–209) in FGF21. The positions at five point-mutations (S195G, P199G, S200G, Q201H, and A208T) are shaded in gray. The area under the curve above the threshold is colored in yellow, while the area below the threshold is shown as green.


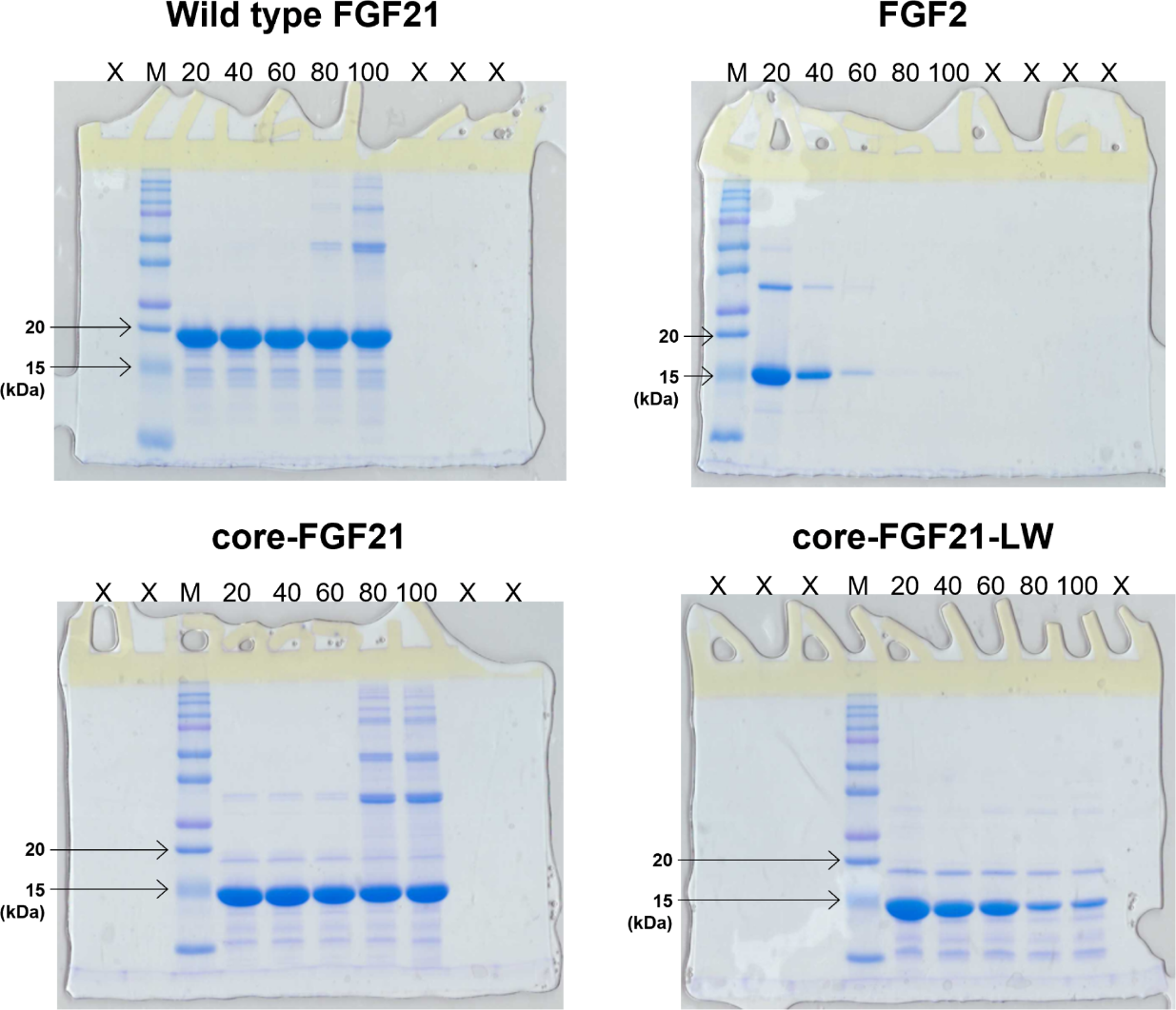


**Fig. S14. Uncropped images of SDS-PAGE showed in Fig. 1A.** Experimental conditions already detailed in main figure and legend.


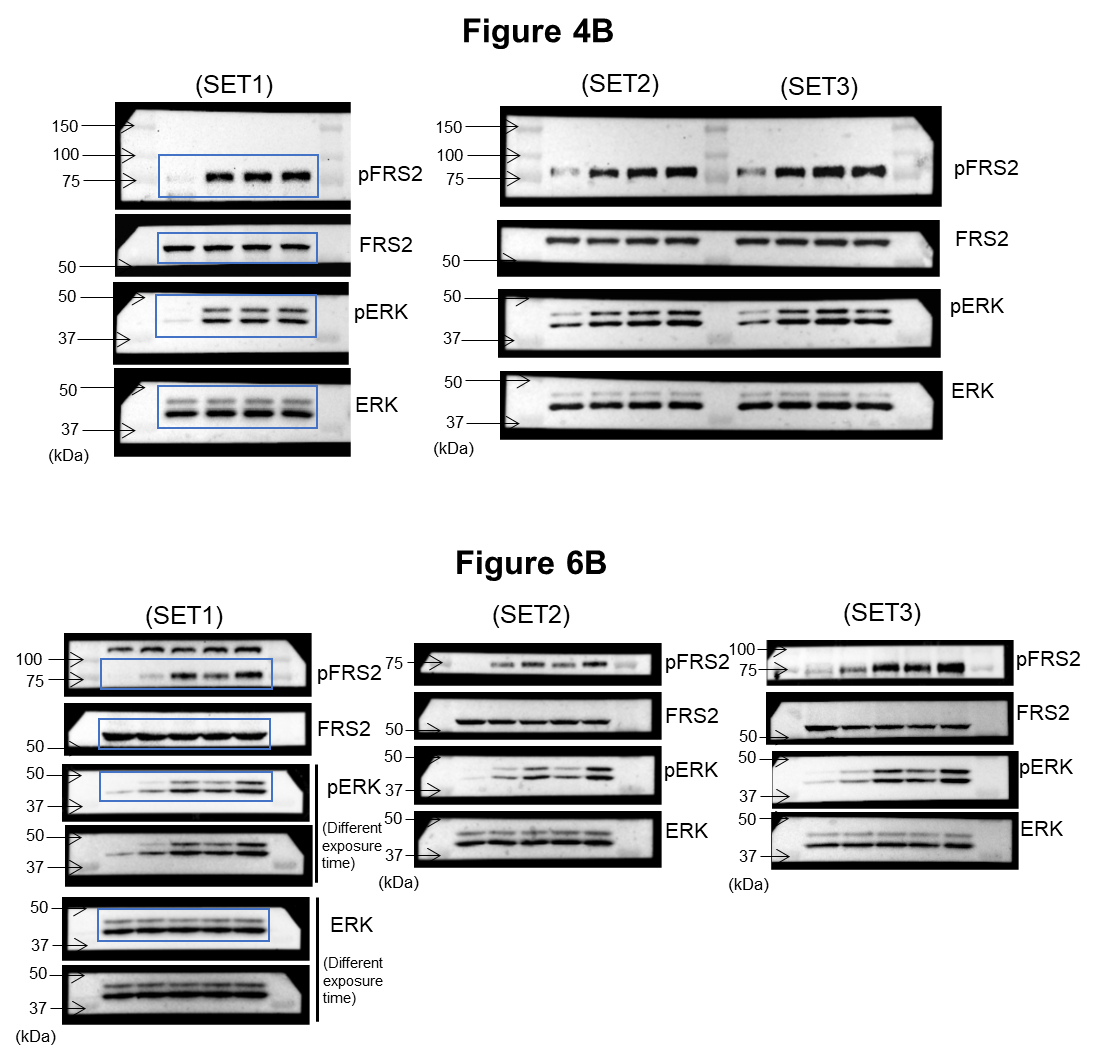


**Fig. S15. Uncropped images of Western-blot analyses showed in Fig. 4B and 6B.** Experimental conditions already detailed in main figures and legends.
